# Flexible Parametric Survival Modeling of Transaminases as Predictive Biomarkers for Non-Alcoholic Fatty Liver Disease: A Retrospective Longitudinal Study (2012–2022)

**DOI:** 10.3390/ijms26115057

**Published:** 2025-05-24

**Authors:** Amr Sayed Ghanem, Ágnes Tóth, Péter Takács, Battamir Ulambayar, Marianna Móré, Attila Csaba Nagy

**Affiliations:** 1 Department of Health Informatics, Faculty of Health Sciences, University of Debrecen, 4028 Debrecen, Hungary; aghanem@etk.unideb.hu (A.S.G.); takacs.peter@etk.unideb.hu (P.T.); ulambayar.battamir@etk.unideb.hu (B.U.); 2 Department of Integrative Health Sciences, Faculty of Health Sciences, University of Debrecen, 4028 Debrecen, Hungary; toth.agnes@etk.unideb.hu; 3 Institute of Social and Sociological Sciences, Faculty of Health Sciences, University of Debrecen, 4400 Nyíregyháza, Hungary; more.mariann@etk.unideb.hu

**Keywords:** non-alcoholic fatty liver disease, NAFLD, GOT, GPT, ALT, AST, Royston–Parmar, flexible parametric survival model

## Abstract

Non-alcoholic fatty liver disease (NAFLD) is a common metabolic liver disease linked to obesity and diabetes. This study aimed to assess whether serum GOT and GPT can predict NAFLD early in at-risk individuals. A retrospective cohort study was conducted using hospital records from the University of Debrecen (2012–2022), including 4886 NAFLD-free individuals at baseline. NAFLD incidence was tracked using ICD-10 codes, with transaminase levels (GOT and GPT) and key metabolic comorbidities analyzed as predictors in a longitudinal design. Survival analysis included Fleming–Harrington tests, Kaplan–Meier, and Nelson–Aalen estimators as well as restricted mean survival time. The Royston–Parmar flexible parametric model was used to assess the time-dependent effects of GOT, GPT, and metabolic risk factors on NAFLD incidence. An elevated GOT was significantly associated with an increased NAFLD hazard (HR = 2.71, 95% CI: 1.31–5.58), as was an elevated GPT (HR = 2.21, 95% CI: 1.09–4.43). Disorders of lipid metabolism showed the strongest association (HR = 3.29, 95% CI: 1.51–7.25). Elevated GOT and GPT levels, in combination with demographic and clinical factors, may serve as valuable prognostic biomarkers for NAFLD progression, underscoring the importance of routine liver enzyme monitoring and comprehensive metabolic management to improve long-term patient outcomes.

## 1. Introduction

Non-alcoholic fatty liver disease (NAFLD) is a chronic liver condition characterized by the accumulation of fat in the hepatocytes in the absence of significant alcohol consumption [1]. New nomenclatures have been proposed to replace NAFLD, aiming to better reflect the metabolic background of the disease: metabolic-dysfunction-associated fatty liver disease (MAFLD) in 2020 and metabolic-dysfunction-associated steatotic liver disease (MASLD) in 2023 [2]. MAFLD introduced positive diagnostic criteria, regardless of alcohol intake or other liver conditions. MAFLD is defined by the presence of hepatic steatosis in combination with at least one of the following criteria: overweight/obesity, type 2 diabetes or the presence of two or more metabolic risk factors [3,4]. MASLD is characterized by hepatic steatosis accompanied by at least one of five cardiometabolic risk factors [5]. In this retrospective longitudinal study spanning the period between 2012 and 2022, we used the term NAFLD for consistency with historical clinical data but acknowledge the shift toward MASLD terminology. NAFLD encompasses two distinct entities: non-alcoholic fatty liver (NAFL), which is generally benign, and non-alcoholic steatohepatitis (NASH), which is marked by inflammation, hepatocellular damage, and a risk of fibrosis progression [1]. These categories are conceptually retained in the updated MASLD framework, where they correspond to MASLD (simple steatosis) and MASH (metabolic-dysfunction-associated steatohepatitis), respectively [6]. 

The global prevalence of NAFLD is estimated to be 25–30%, affecting over 1.8 billion individuals worldwide, with significant regional variation. The highest prevalence rates are observed in the Middle East (32%) and South America (30%), followed by North America (24%) and Asia (27%) [7,8]. Europe has a prevalence rate of approximately 24%, with trends driven by rising obesity and metabolic syndrome rates [9]. In 2021, the global age-standardized incidence rate was 592.78 per 100,000 population (95% CI: 542.23–643.24), with a mortality rate of 1.14 per 100,000 (95% CI: 0.82–1.52) [10]. In the European Union, the incidence rate was 411.88 per 100,000 (95% CI: 378.89–446.40), and the mortality rate was 1.41 per 100,000 (95% CI: 1.00–1.86) [10]. In Hungary, rates were higher, with an incidence rate of 463.31 per 100,000 (95% CI: 424.18–502.10) and a mortality rate of 1.45 per 100,000 (95% CI: 0.94–2.15) [10].

NAFLD is closely linked to metabolic disorders, making it a hepatic manifestation of metabolic syndrome [11]. It was shown that patients with metabolic syndrome had a substantially increased likelihood of developing hepatic steatosis and fibrosis, with risk estimates ranging from 34 to 36% and 42 to 47%, respectively, depending on diagnostic criteria [12]. NAFLD affects 55–70% of individuals with T2DM, associated with an increased risk of fibrosis development [13], and it has been also strongly associated with hypertension, dyslipidemia [14], overweight/obesity, and IR [15]. IR plays a crucial role in NAFLD development [16], with baseline IR being linked to the progression of liver fibrosis in patients without diabetes [17]. C-reactive protein (CRP), a marker of inflammation, has been found to be elevated in both NAFLD and NASH [18] and may therefore be considered an important risk factor [19]. Beyond liver-related morbidity and mortality, NAFLD is also associated with an increased risk of cardiovascular disease, extra-hepatic cancers, and chronic kidney disease [20].

At the molecular level, disruptions in lipid metabolism and mitochondrial dysfunction are central to NAFLD progression. The suppression of fatty acid β-oxidation, mediated by reduced peroxisome-proliferator-activated receptor-alpha (PPAR-α) activity [21], contributes to lipid accumulation and hepatocellular stress. PPAR-α regulates the expression of key enzymes, including carnitine palmitoyltransferase 1 (CPT1) and medium-chain acyl-CoA dehydrogenase (MCAD), which are essential for fatty acid oxidation [22]. Impaired mitochondrial function further exacerbates oxidative stress and promotes hepatocellular injury, creating a feedback loop that accelerates disease progression [23].

Alanine aminotransferase (ALT) and aspartate aminotransferase (AST), also known as glutamate-pyruvate transaminase (GPT) and glutamate-oxaloacetate transaminase (GOT), respectively, are widely used biomarkers of liver health. Recent studies suggest that elevations in aminotransferases reflect the metabolic demands placed on the liver rather than simple leakage due to cell injury [24,25]. This hypothesis is supported by evidence showing associations between serum aminotransferase levels and markers of metabolic dysfunction, including triglycerides, fasting glucose, and high body mass index [26,27,28]. In some cases, aminotransferases have been proposed as part of diagnostic panels for steatohepatitis and fibrosis [29,30], although their sensitivity and specificity remain variable. Elevated GPT levels, even within normal ranges, have been linked to a higher risk of NAFLD and associated complications [31], though a consensus on cutoff values for GPT remains elusive.

This study aimed to evaluate the predictive utility of serum aminotransferases (GOT and GPT) for non-alcoholic fatty liver disease in the context of metabolic risk factors, with an emphasis on early identification before irreversible liver damage occurs. The GOT/GPT pair offer a cost-effective and widely accessible option for screening at -risk populations, potentially preventing disease progression and the associated comorbidities.

## 2. Results

The study sample at baseline (*n* = 4886) consisted of 38.99% individuals under 45 years of age, 45.58% aged 45–64, and 15.43% aged 65 or older outlined in Table 1. Women made up 54.58% of the cohort, while men accounted for 45.42%. Elevated GOT levels were observed in 12.12% (*n* = 64) of the 528 participants assessed, and elevated GPT levels were noted in 13.45% (*n* = 76) of the 565 evaluated. Among the 438 participants with CRP measurements, 18.26% (*n* = 80) had levels > 15. Essential hypertension was present in 12.53% (*n* = 612) of the cohort. The prevalence of angina pectoris, chronic ischemic heart disease, and heart failure was 6.88%, 3.50%, and 1.27%, respectively. Atherosclerosis affected 1.96%, while type 2 diabetes mellitus and obesity were present in 2.07% and 2.42% of individuals, respectively. Finally, disorders of lipid metabolism were identified in 6.02% (*n* = 294).

**Figure 1:**
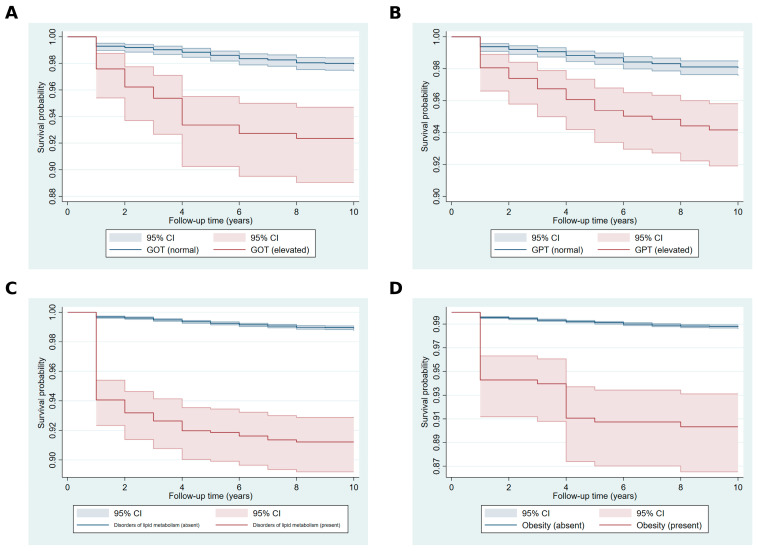
Kaplan–Meier survival curves for key covariates. Footer: (**A**) GOT (glutamic oxaloacetic transaminase): blue line for normal, red line for elevated. (**B**) GPT (glutamic pyruvic transaminase): blue line for normal, red line for elevated. (**C**) Disorders of lipid metabolism: blue line for absent, red line for present. (**D**) Obesity: blue line for non-obese, red line for obese. Shaded areas represent 95% confidence intervals.

The Kaplan–Meier survival curves depicted in Figure 1 show the survival probabilities over the follow-up period for individuals stratified by key covariates. Elevated GOT and GPT levels were associated with reduced survival probabilities compared to normal levels, with differences becoming more pronounced in later follow-up years (Figure 1A,B). Individuals with lipid metabolism disorders exhibited consistently lower survival probabilities compared to those without such disorders (Figure 1C). Similarly, obesity was linked to a notable decrease in survival probabilities, with the gap between obese and non-obese groups widening over time (Figure 1D).

The Fleming–Harrington test (parameters *ρ* = 0, *γ* = 1, emphasizing later events) revealed significant differences in survival distributions across the various subgroups, shown in Table 2. Elevated GOT (*p* < 0.001) and GPT (*p* < 0.001) were strongly associated with increased event rates. Significant differences were also observed for age groups (*p* = 0.012), sex (*p* = 0.014), essential hypertension (*p* = 0.007), chronic ischemic heart disease (*p* = 0.005), heart failure (*p* < 0.001), type 2 diabetes mellitus (*p* = 0.005), obesity (*p* < 0.001), and disorders of lipid metabolism (*p* < 0.001). No significant differences were found for C-reactive protein (*p* = 0.251), angina pectoris (*p* = 0.858), or atherosclerosis (*p* = 0.278).

**Figure 2:**
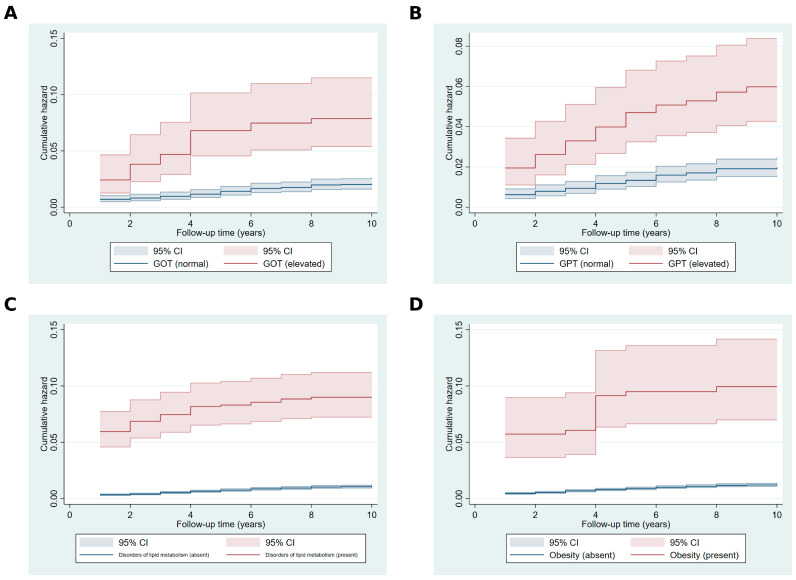
Nelson–Aalen cumulative hazard estimates for key covariates. **Footer**: (**A**) GOT (glutamic oxaloacetic transaminase): blue line for normal, red line for elevated. (**B**) GPT (glutamic pyruvic transaminase): blue line for normal, red line for elevated. (**C**) Disorders of lipid metabolism: blue line for absent, red line for present. (**D**) Obesity: blue line for non-obese, red line for obese. Shaded areas represent 95% confidence intervals.

The Nelson–Aalen cumulative hazard estimates illustrated in Figure 2 display the accumulation of hazard over the follow-up period for key covariates. Participants with elevated GOT and GPT levels showed substantially higher cumulative hazard rates compared to those with normal levels, with divergence becoming more apparent as follow-up time increased (Figure 2A,B). Similarly, individuals with disorders of lipid metabolism exhibited a steeper accumulation of hazard compared to those without such disorders (Figure 2C). Obesity was associated with a significantly greater cumulative hazard, with the gap between participants with and without obesity widening consistently throughout the follow-up period (Figure 2D).

Restricted mean survival time (RMST) estimates displayed in Table 3 showed significant differences across various groups. In the age groups, those aged 45–64 had an RMST of 6.944 years (95% CI: 6.938–6.950), 0.022 years lower than those <45 years (*p* < 0.001), while those aged 65+ had an RMST of 6.952 years (95% CI: 6.946–6.959), 0.013 years lower than those <45 (*p* = 0.004). Sex analysis revealed that men had an RMST of 6.939 years (95% CI: 6.933–6.945), 0.022 years lower than women (*p* < 0.001). Elevated GOT levels were associated with a significantly reduced RMST of 6.686 years (95% CI: 6.582–6.791), 0.247 years lower than normal levels (*p* < 0.001). Similarly, elevated GPT levels reduced the RMST to 6.787 years (95% CI: 6.726–6.847), 0.149 years lower than normal levels (*p* < 0.001). High C-reactive protein levels resulted in an RMST of 6.893 years (95% CI: 6.846–6.939), 0.055 years lower than normal levels (*p* = 0.027). Essential hypertension, angina pectoris, chronic ischemic heart disease, and type 2 diabetes mellitus were all associated with significantly reduced RMSTs by 0.22 years (*p* < 0.001), 0.237 years (*p* < 0.001), 0.245 years (*p* < 0.001), and 0.263 years (*p* < 0.001), respectively. Heart failure (tau = 5) reduced the RMST by 0.079 years to 4.895 years (95% CI: 4.866–4.924, *p* < 0.001). Atherosclerosis (tau = 3) resulted in an RMST reduction of 0.111 years to 2.88 years (95% CI: 2.880–2.880, *p* < 0.001). Obesity was associated with the largest reduction, lowering the RMST by 0.406 years to 6.551 years (95% CI: 6.467–6.634, *p* < 0.001). Disorders of lipid metabolism decreased the RMST by 0.412 years to 6.553 years (95% CI: 6.504–6.602, *p* < 0.001).

**Figure 3:**
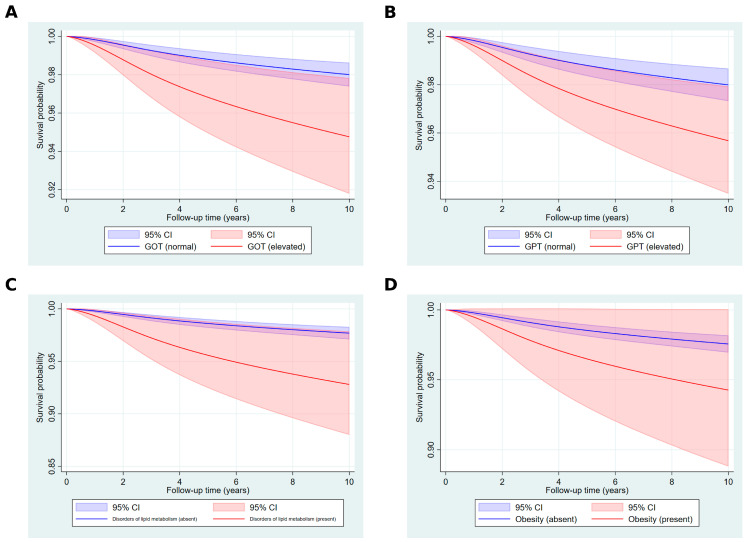
Standardized survival curves for GOT, GPT, disorders of lipid metabolism, and obesity over a 10-year follow-up period. **Note**: The figure shows the standardized survival probabilities for four key covariates: (**A**) GOT (normal: blue line and shaded area; elevated: red line and shaded area), (**B**) GPT (normal: blue line and shaded area; elevated: red line and shaded area), (**C**) disorders of lipid metabolism (absent: blue line and shaded area; present: red line and shaded area), and (**D**) obesity (non-obese: blue line and shaded area; obese: red line and shaded area). Shaded areas represent 95% confidence intervals.

Figure 3 presents the standardized survival curves for key covariates, highlighting the differences in survival probabilities over a 10-year follow-up period. For the GOT levels, individuals with an elevated GOT had markedly lower survival probabilities compared to those with a normal GOT, with a divergence becoming more pronounced over time (Figure 3A). A similar pattern was observed for the GPT levels, where an elevated GPT was associated with reduced survival probabilities compared to a normal GPT (Figure 3B). For disorders of lipid metabolism, individuals with the condition exhibited significantly lower survival probabilities throughout the follow-up compared to those without (Figure 3C). Finally, individuals with obesity demonstrated consistently lower survival probabilities compared to those without obesity, with a noticeable gap widening over the follow-up period (Figure 3D).

In the flexible parametric survival model, an elevated GOT was significantly associated with an increased hazard of NAFLD incidence as shown in Table 4, with a hazard ratio (HR) of 2.71 (95% CI: 1.31–5.58, *p* = 0.007). Similarly, an elevated GPT was linked to an increased hazard, with an HR of 2.21 (95% CI: 1.09–4.43, *p* = 0.027). Disorders of lipid metabolism were strongly associated with an increased hazard of NAFLD, with an HR of 3.29 (95% CI: 1.51–7.25, *p* = 0.003). The nonlinear spline effects showed a significant time-dependent pattern of hazard variation, with _rcs1 having a HR of 1.43 (95% CI: 1.32–1.57, *p* < 0.001) and _rcs2 a HR of 1.06 (95% CI: 1.00–1.12, *p* = 0.039). Other covariates, including age, sex, C-reactive protein, essential hypertension, angina pectoris, chronic ischemic heart disease, heart failure, atherosclerosis, type 2 diabetes mellitus, and obesity, showed no statistically significant association with the hazard of NAFLD incidence (all *p* > 0.05).

**Figure 4:**
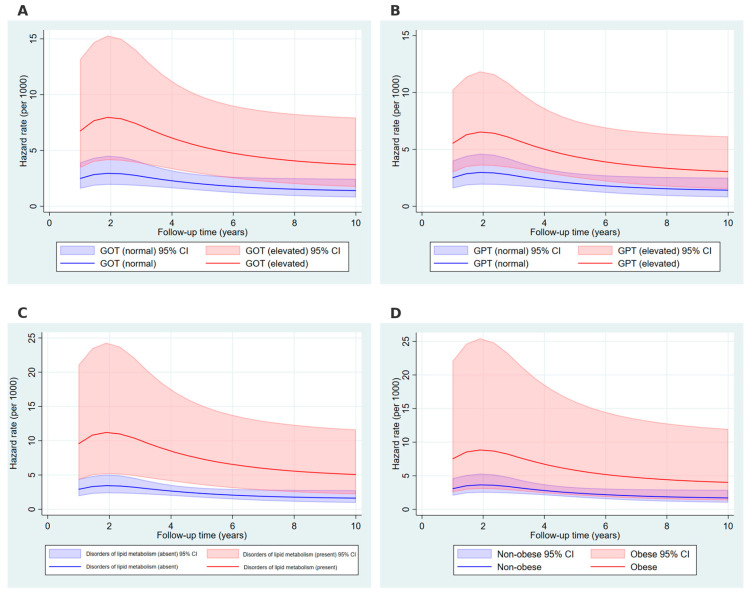
Hazard rates over time for key covariates. **Footer**: GOT (glutamate oxaloacetate transaminase) and GPT (glutamate pyruvate transaminase) hazard rates are shown in blue and red lines, with normal values (blue solid line, shaded CI) and elevated values (red solid line, shaded CI) for each covariate. Panels: (**A**) GOT, (**B**) GPT, (**C**) disorders of lipid metabolism, (**D**) obesity. Shaded regions represent 95% confidence intervals, with elevated covariate levels consistently associated with higher hazard rates and wider intervals due to smaller sub-sample sizes.

Figure 4 illustrates the time-dependent hazard rates (per 1000 person-years) for the development of non-alcoholic fatty liver disease across a 10-year follow-up period. Elevated GOT and GPT levels were associated with consistently higher hazard rates compared to normal levels, peaking within the first two years and gradually declining thereafter (Figure 4A,B). Similarly, individuals with disorders of lipid metabolism exhibited significantly higher hazard rates compared to those without such disorders, with a notable peak early in the follow-up period (Figure 4C). Obesity was also associated with markedly increased hazard rates relative to individuals without obesity, particularly in the initial follow-up period, followed by a gradual decrease over time (Figure 4D). The shaded regions represent the 95% confidence intervals, with elevated covariate levels consistently associated with higher hazard rates and wider intervals due to smaller sub-sample sizes.

## 3. Discussion

Non-alcoholic fatty liver disease (NAFLD) is a serious and emerging health concern globally [32,33]. In this study, we investigated the role of transaminases, specifically GOT (also called AST) and GPT (also called ALT), as potential predictive biomarkers for the development and progression of the disease. We utilized Kaplan–Meier survival curves, standardized survival curves, and the Flaming–Harrington test to assess survival probabilities and compare survival distributions across different clinical subgroups. Additionally, we calculated restricted mean survival times and used flexible parametric survival models to further quantify the prognosis of patients with NAFLD. Furthermore, we employed Nelson–Aalen cumulative hazard curves to evaluate these survival distributions, and we determined the hazard rates over time. These analyses allowed us to examine the relationship of transaminase levels and other factors with the progression of NAFLD, providing valuable insights into the potential of these biomarkers in predicting disease outcomes.

In our analysis, the Kaplan–Meier survival curves showed associations between elevated GOT and GPT levels with reduced survival probabilities compared to normal levels. Accordingly, individuals with elevated GOT and GPT levels had markedly lower survival probabilities compared to those with normal GOT and GPT levels as reflected in the standardized survival curves. In both types of curves, the differences became more pronounced in subsequent years of follow-up. The Nelson–Aalen cumulative hazard estimates demonstrated significantly higher cumulative hazard rates for patients with elevated GOT and GPT levels compared to those with normal enzyme levels. The divergence between these two groups became more pronounced over the follow-up period, suggesting that elevated transaminase levels correlate with a higher risk of adverse health outcomes as NAFLD progresses. Based on our time-dependent hazard rates over the 10-year follow-up period for NAFLD development, we found associations between elevated GOT and GPT levels with consistently higher hazard rates compared to normal levels, reaching a peak in the first two years, followed by a progressive drop. Our graphical results are consistent with our quantitative and parametric analyses. The Fleming–Harrington test, which examines survival distributions in different subgroups of patients with NAFLD, revealed that elevated GOT and GPT levels were strongly associated with increased event rates. The restricted mean survival time (RMST) estimates showed that elevated GOT and GPT were associated with a significantly reduced RMST compared to normal levels. Furthermore, we investigated their association with the risk of NAFLD incidence, and we found that elevated levels of these liver enzymes were strongly linked to the incidence of the disease.

These results suggest that higher transaminase levels are indicators of worse health outcomes, likely pointing to more severe liver dysfunction or the progression of NAFLD. The progressive nature of these findings may indicate that the longer the follow-up period, the clearer the impact of elevated transaminases becomes on survival. This could imply that elevated enzyme levels contribute to a worsening risk of mortality or other health complications related to liver disease, and this risk becomes more evident as the disease advances. Our findings are consistent with previous findings that show that elevated levels of liver enzymes (including ALT and AST) are associated with increased liver disease mortality [34], and that patients with NAFLD and elevated liver enzyme levels (ALT and AST levels) display significantly higher risk for cirrhosis and hepatocellular carcinoma [35]. It was also demonstrated that elevated ALT levels were associated with increased cardiovascular disease (CVD)- or diabetes-related mortality [36].

Elevated transaminases may not be the only markers of poor prognosis in NAFLD. Disorders in lipid metabolism could also play a critical role in predicting patient outcomes. The Kaplan–Meier survival curves showed that the survival of individuals with lipid metabolism disorders was significantly affected, as indicated by the consistently lower survival probability compared to those without such disorders. In accordance with this, the standardized survival curves also showed a significantly lower survival probability for individuals with lipid metabolism disorders compared to individuals without the disease throughout the follow-up period. When analyzing the cumulative hazard of patients with NAFLD utilizing Nelson–Aalen cumulative hazard estimates, we found significantly higher rates in the case of individuals with disorders of lipid metabolism compared to those without such conditions. Similarly, the time-dependent hazard rate analyses revealed that individuals with lipid metabolism disorders had significantly higher hazard rates compared to those without such disorders. Our statistical analyses, including the Fleming–Harrington test, revealed that there was a strong association between disorders of lipid metabolism with increased event rates. In addition, we found that this medical condition corresponded with lower RMSTs. The flexible parametric survival model revealed that these types of disorders were strongly associated with an increased risk of developing NAFLD. 

The results suggest that individuals with lipid metabolism disorders face a higher risk of adverse outcomes, which contribute to a worse prognosis (such as disease progression or death) for NAFLD patients compared to those without these disorders, leading to higher risk and lower survival rates. Comprehensive clinical reviews have concluded that NAFLD often coexists with the components of metabolic syndrome, including dyslipidemia, contributing to the significantly lower survival rates compared to those without NAFLD [37]. It has been demonstrated that the excessive accumulation of lipids, primarily triglycerides, is closely linked to the metabolic dysfunction characteristic of NAFLD. This accumulation exacerbates other conditions, including insulin resistance, leading to an elevated risk of liver damage and the progression of the disease [38]. In addition, dysregulated lipid metabolism links NAFLD to cardiovascular disease [39]. 

Obesity may also have a significant impact on the survival and hazard of patients with NAFLD. As the Kaplan–Meier survival curves indicated, we found a notable decrease in survival probabilities in patients with compared with patients without obesity. The gap in survival probabilities between the obese and non-obese groups widened over the follow-up period, further emphasizing the negative impact of obesity on NAFLD prognosis. Similarly, individuals with obesity displayed consistently lower survival probabilities based on our standardized survival curves compared to those without obesity, again, with a gap widening over time. According to the Nelson–Aalen cumulative hazard estimates, obesity was linked to a noticeably higher cumulative hazard, and the difference between participants with and without obesity steadily increased over the course of the follow-up period. Similar results were obtained from the time-dependent hazard rates, which showed that obesity was associated with significantly higher hazard rates compared with non-obesity, particularly during the initial follow-up period. The Fleming–Harrington test showed significant associations between obesity and increased event rates. Examining the RMSTs, obesity, in particular, yielded the largest reduction in the RMST in different subgroups of patients with NAFLD, further emphasizing its role as a critical modifiable risk factor for NAFLD progression and the associated mortality, in accordance with previous findings [40].

The results underscore the significant impact of obesity. Apart from elevated levels of liver enzymes and disorders of lipid metabolism, it was also strongly associated with increased hazard and lower survival rates, not only elevating risks but also significantly reducing long-term survival chances. It has been shown that besides dyslipidemia, NAFLD is frequently accompanied by other components of metabolic syndrome, such as obesity, to lower survival rates compared to individuals without NAFLD [37]. patients with overweight or obesity with NAFLD tend to have more severe histological features, predicting a worse long-term prognosis [41] including the development of cirrhosis and hepatocellular carcinoma. As a result, liver-specific mortality is significantly higher in patients with NAFLD [40].

Our statistical approaches demonstrate that besides the GOT and GPT levels, disorders of lipid metabolism, and obesity, certain other factors, such as demographic characteristics, clinical parameters, and different comorbidities may be critical determinants of survival as well. Factors such as age and sex were also significantly associated with increased event rates and lower RMSTs. Adults aged 45–64 demonstrated a lower RMST compared to younger (<45 years) and older (>65 years) counterparts. This could be attributed to a combination of age-related metabolic changes and the increased prevalence of comorbid conditions during this life stage. The sex analysis aligns with the existing literature suggesting that men experience more severe forms of NAFLD and its complications. The prevalence and severity of NAFLD are higher in men than in women during reproductive age, suggesting that estrogen is protective [42]. Cardiovascular conditions, such as essential hypertension [43,44], atherosclerosis [45,46], angina pectoris, chronic ischemic heart disease [47], and heart failure [39,48,49], all correlate with increased event rates and/or lower RMST values, underscoring earlier findings. Numerous mechanisms, including systemic inflammation, endothelial dysfunction, hepatic insulin resistance, oxidative stress, and altered lipid metabolism, have been identified as pathways through which NAFLD increases the risk of cardiovascular disease [46]. Type 2 diabetes mellitus (T2DM) shows increased event rates and lower RMST values. T2DM and NAFLD frequently exist together [50]. A significant majority of individuals with type 2 diabetes are overweight or obese [51]. Overweight/obesity and insulin resistance (IR) have been strongly linked with NAFLD [15]. The interplay between fat accumulation, insulin resistance, increased oxidative stress, and inflammation generates a pathway that impairs both liver and metabolic health [52]. An elevated C-reactive protein (CRP) level corresponds to a significantly reduced RMST. The relationship suggests a link between liver function and systemic inflammation. Hepatic steatosis may enhance the secretion of high-sensitivity CRP (hs-CRP) in the liver, elevating systemic hs-CRP levels; however, the inflammatory response is likely to act as a feedback loop that contributes to hepatic steatosis and systemic changes, which increase the risk of cardiovascular disease [53].

This study has several limitations inherent to the retrospective nature of the dataset. First, the diagnosis of NAFLD was based on ICD-10 codes, without access to the specific diagnostic modality used, precluding assessments of diagnostic sensitivity and specificity. However, coding was performed by physicians as part of routine clinical care, aligning with standard practice in registry-based research. Second, serum transaminase and CRP values were available only for a subset of patients, which may have introduced selection bias; nonetheless, complete-case analyses and sensitivity checks confirmed that the main findings were robust. Third, information on alcohol consumption, which is essential for excluding other causes of fatty liver, was not available, limiting the ability to verify the diagnostic context of NAFLD. This is especially relevant given the increasingly recognized overlap and shared mechanisms between alcohol-related liver disease (ALD) and NAFLD, as highlighted in the recent literature [54]. Additionally, the dataset lacked key lifestyle and behavioral covariates such as smoking, diet, physical activity, and medication use, which may have contributed to residual confounding. The single-center design may also constrain generalizability, though it ensures consistency in data recording and clinical protocols. Lastly, as in most retrospective database studies, the validity of ICD coding could not be independently verified, and misclassification bias cannot be entirely excluded.

## 4. Materials and Methods

### 4.1. Data Cleaning and Processing

This retrospective longitudinal study utilized clinical data from hospital records at the Clinical Center of the University of Debrecen, spanning the period between 2007 and 2022. However, due to missing and inconsistent data quality, records prior to 2012 were excluded. The study baseline was set to 2012, ensuring that individuals with pre-existing diagnoses of NAFLD were excluded, establishing a cohort free of the disease at baseline. The failure event was defined as the diagnosis of NAFLD, identified using ICD-10 codes. The final dataset comprised a total of 29,903 observations, representing 4886 unique individuals. Among total observations, 495 were indicative of NAFLD diagnosis during the follow-up period. For participants diagnosed with NAFLD, the year of diagnosis marked the end of follow-up, while for censored participants, follow-up extended to either the last year of available data or the study endpoint in 2022.

### 4.2. Variables of Interest

Age was categorized into three groups: <45 years, 45–64 years, and ≥65 years. Sex was dichotomized as male or female. Biomarkers of interest included C-reactive protein (CRP), with a cutoff of 15 mg/L to define high levels, and transaminases (GOT and GPT), which were classified as elevated if values exceeded 40 IU/L. Cutoffs for transaminases were determined based on median laboratory measurements for each year to account for potential variability in clinical practice, providing a robust epidemiological basis. Disease diagnoses were treated as binary variables (present vs. absent) and identified using ICD-10 codes, including essential hypertension (I10), angina pectoris (I20), chronic ischemic heart disease (I25), heart failure (I50), atherosclerosis (I70), type 2 diabetes mellitus (E11), obesity (E66), and disorders of lipid metabolism (E78).

### 4.3. Inclusion and Exclusion Criteria

To establish a NAFLD-free cohort at baseline, individuals with a documented NAFLD diagnosis before 2012 were excluded, preventing prevalence bias. Participants were included if they had at least two recorded hospital encounters and a minimum follow-up of one year to ensure adequate observation time for survival analysis.

Exclusion criteria encompassed individuals with incomplete or inconsistent diagnostic records for key covariates, particularly comorbid conditions such as hypertension, diabetes, obesity, and lipid disorders, to minimize misclassification bias. Individuals with only a single recorded hospital visit were also excluded due to insufficient follow-up, mitigating immortal time bias.

Participants without NAFLD by this study’s end were censored at their last recorded visit or the 2022 endpoint. Loss to follow-up was assumed to be non-informative, meaning it was independent of NAFLD development risk. This approach ensured a well-defined cohort with robust exposure and outcome data for longitudinal analysis while addressing potential biases inherent in electronic health records.

### 4.4. Statistical Analysis

Baseline characteristics were assessed using the initial recorded data for each participant in the dataset. All variables, being categorical in nature, are described using frequencies and percentages.

#### 4.4.1. Fleming–Harrington Test and Visualizations

The Fleming-Harrington test [55], a weighted log-rank test, was employed for univariate analysis to evaluate survival differences across the categories of each variable in the dataset. This test uses a weighting scheme defined by parameters (ρ, γ), where ρ = 0 and γ = 1 in this study placed greater emphasis on later events during follow-up. This weighting approach is particularly useful in scenarios where late failures are of interest, as it assigns higher weights to observations occurring toward the end of the follow-up period.

The test evaluates whether survival curves differ significantly between groups and provides a *p*-value as a measure of statistical significance. The test outputs include observed and expected events, the sum of ranks, and the associated *p*-value, which reflect the contribution of each variable’s categories to the risk of developing NAFLD. This method aids in identifying variables with significant survival differences, highlighting their potential association with NAFLD incidence.

The formula for the Fleming-Harrington test statistic is as follows:

(1) $$Z=\frac{\sum_{i=1}^{n}w(t_{i})(O_{i}-E_{i})}{\sqrt{\sum_{i=1}^{n}w(t_{i}{)}^{2}Var(O_{i})}}$$

where:

$w\left(t_{i}\right)=Ŝ(t_{i}{)}^{\rho }(1-Ŝ(t_{i}){)}^{\gamma }$: the weight function based on the Kaplan-Meier estimate $Ŝ(t_{i})$ of the survival probability at time $t_{i}$, with ρ and γ defining the weight’s dependence on survival probabilities.

$O_{i}$: the observed number of events at time $t_{i}$.

$E_{i}$: the expected number of events at time $t_{i}$ under the null hypothesis.

$Var(O_{i})$: the variance of the observed events at $t_{i}$.

This test is particularly suited for identifying survival differences influenced by variables that may affect late outcomes, such as disease progression or delayed onset of events.

The Kaplan-Meier survival curve [56] is a non-parametric method used to estimate survival probabilities over time, accounting for censored data. It provides a stepwise representation of survival, with drops occurring at each failure event, making it particularly useful for visualizing differences in survival across groups. The survival probability at time t is calculated as:

(2) $$Ŝ\left(t\right)=\prod_{t_{i}\leq t}\left(1-\frac{d_{i}}{n_{i}}\right)$$

where:

$Ŝ\left(t\right)$ is the estimated survival probability at time t,

$d_{i}$ is the number of events (diagnosis of NAFLD) at time $t_{i}$,

$n_{i}$ is the number of participants at risk just before time $t_{i}.$

The Nelson-Aalen cumulative hazard estimator [57] is a non-parametric method used to estimate the cumulative hazard function over time. It is particularly useful for understanding the accumulation of risk for an event, such as disease onset, in a population while accounting for censored data. Unlike the Kaplan-Meier estimator, which focuses on survival probabilities, the Nelson-Aalen estimator provides insights into the rate at which the hazard accumulates, offering a complementary perspective for survival analysis.

The cumulative hazard at time t is calculated as:

(3) $$\hat{H}\left(t\right)=\sum_{t_{i}\leq t}\frac{d_{i}}{n_{i}}$$

where:

$\hat{H}\left(t\right)$ is the cumulative hazard estimate and time t,

$t_{i}$ denotes the observed event times,

$d_{i}$ is the number of events (NAFLD diagnoses) at $t_{i}$,

$n_{i}$ is the number of individuals at risk just prior to $t_{i}$.

The Nelson-Aalen estimator is robust for identifying how risk accumulates over time and is particularly valuable for comparing cumulative hazards between groups. This rationale makes it suitable for the presented analysis, where elevated GOT, GPT, disorders of lipid metabolism, and obesity are evaluated for their contributions to cumulative hazard trends over the study period.

#### 4.4.2. Restricted Mean Survival Time Analysis and Standardized Survival Curves

The restricted mean survival time (RMST) is a comprehensive metric in survival analysis that estimates the average time until an event occurs [58], restricted to a specified time horizon τ. Unlike the median survival time or hazard ratios, RMST utilizes the entire survival curve up to τ, offering a clinically interpretable and robust summary measure of survival differences between groups. This makes RMST particularly useful when the proportional hazards assumption does not hold or when comparing groups with incomplete follow-up.

RMST is mathematically defined as:

(4) $$RMST\left(\tau \right)=\int_{0}^{\tau }Ŝ\left(t\right)\;dt$$

where:

$Ŝ\left(t\right)$: the Kaplan-Meier estimate of the survival probability at time t,

τ: the time horizon up to which survival probabilities are integrated.

The formula calculates the area under the survival curve up to the specified time horizon τ, providing the mean survival time for a population within the defined follow-up period. Differences in RMST between groups quantify the survival advantage or disadvantage attributable to specific covariates.

RMST was suitable for evaluating the impact of key covariates, such as GOT, GPT, disorders of lipid metabolism, and obesity, on survival outcomes. Its ability to account for the entire survival curve and provide clinically meaningful comparisons aligns with the goals of the analysis, especially in the presence of time-limited follow-up data and non-proportional hazards.

Standardized survival curves provide an adjusted estimate of survival probabilities for a population, accounting for the distribution of covariates. These curves are derived by averaging individual survival probabilities predicted from a model, such as a Cox proportional hazards model or a flexible parametric survival model, over the covariate distribution in the study population. This standardization process allows for a comparison of survival outcomes across groups while controlling for confounding variables, making them particularly useful in epidemiological and clinical research.

The standardized survival probability at time t is calculated as:

(5) $${Ŝ}_{std}\left(t\right)=\frac{1}{n}\sum_{i=1}^{n}{Ŝ}_{i}(t)$$

where:

${Ŝ}_{std}\left(t\right)$: the standardized survival probability at time t,

${Ŝ}_{i}(t)$: the predicted survival probability for individual i at time t,

n: the total number of individuals in the population.

This formula computes the mean of the predicted survival probabilities for all individuals in the dataset, reflecting the survival probability for a population with an average covariate distribution.

In this study, standardized survival curves were employed to evaluate survival differences for key covariates, including GOT, GPT, disorders of lipid metabolism, and obesity. These curves provide a clear visual representation of the survival experience of the population, adjusted for confounding factors, ensuring robust and interpretable comparisons across groups.

#### 4.4.3. Flexible Parametric Survival Modeling and Time Dependent Hazard Rate Curves

The Royston–Parmar model [59] is an advanced statistical method used to analyze time-to-event data, while allowing for greater flexibility in modeling the baseline hazard function. This approach extends the traditional Cox proportional hazards model by using restricted cubic splines to model the log baseline cumulative hazard, enabling it to capture nonlinear trends in the data. Its flexibility makes it well suited for situations where the proportional hazards assumption may not hold or where complex survival patterns need to be modeled.

The final Royston–Parmar model in this study was selected by manually adjusting the number and placement of spline knots to achieve the best fit, as evaluated using Akaike Information Criterion (AIC) and Bayesian Information Criterion (BIC). The chosen model utilized three knots to balance flexibility and parsimony, with the hazard function used as the scale parameter to account for time-dependent effects.

This final model provided an optimal fit for the data, enabling the assessment of both linear and non-linear effects of covariates, such as GOT, GPT, and other clinical factors, on the risk of NAFLD. This approach ensured a robust analysis while accommodating potential complexities in the hazard function.

The formula for the flexible parametric survival model (FPM) is expressed as follows:

(6) $$\log\;H\left(t|X\right)=\beta_{0}+\sum_{k=1}^{K}\beta_{k}.Z_{k}\left(t\right)+\sum_{j=1}^{p}\gamma_{j}.X_{j}$$

where:

$H\left(t|X\right)$: the hazard function at time t for covariates X,

$\beta_{0}$: the intercept term,

$Z_{k}\left(t\right)$: the restricted cubic spline terms for modeling the baseline cumulative hazard function, where K is the number of knots,

$\beta_{k}$: coefficients associated with the spline terms,

$\gamma_{j}$: coefficients for the covariates $X_{j}$,

$X_{j}$: covariates included in the model, such as GOT, GPT, and other clinical variables,

p: the total number of covariates in the model.

The restricted cubic splines $Z_{k}\left(t\right)$ allow the model to capture non-linear trends in the log cumulative hazard function, providing greater flexibility than traditional models.

Time-dependent hazard rate curves provide a dynamic representation of the instantaneous risk of an event (NAFLD diagnosis) over time, conditional on survival up to that point. Unlike cumulative measures, hazard rate curves capture temporal variations in risk, offering detailed insights into how the likelihood of an event changes during follow-up. These curves are particularly useful in identifying periods of elevated or declining risk and are often derived from flexible parametric survival models for greater accuracy and interpretability. These curves were generated to evaluate the evolving risk of NAFLD associated with key covariates (GOT, GPT, disorders of lipid metabolism, and obesity). These curves revealed how the risk fluctuates over time, with distinct patterns observed for elevated biomarker levels and clinical conditions. This approach provided a detailed understanding of temporal risk dynamics, showcasing periods of peak hazard and the long-term impact of the studied factors.

The hazard rate at time t is defined as:

(7) $$h\left(t|X\right)=\frac{f\left(t|X\right)}{S\left(t|X\right)}$$

where:

$h\left(t|X\right)$: the hazard rate at time t given covariates X,

$f\left(t|X\right)$: the probability density function of the time-to-event for covariates X,

$S\left(t|X\right)$: the survival function, representing the probability of surviving beyond time t.

Using the flexible parametric survival model, the log cumulative hazard is modelled with splines, and the hazard rate is obtained by differentiating the cumulative hazard function with respect to time.

Due to the nature of routine clinical data collection, serum transaminases (GOT, GPT) and C-reactive protein (CRP) were not available for all patients in the cohort. For models involving these variables, complete-case analysis was applied, restricting the sample to individuals with non-missing values for the biomarkers of interest. This approach is commonly employed in retrospective observational studies where imputation may be inappropriate, particularly for complex, nonlinearly distributed biomarkers that lack strong predictors among the available covariates. To assess the potential for bias introduced by this approach, sensitivity analyses was conducted comparing model estimates with and without the inclusion of GOT, GPT, and CRP. The results remained consistent in direction and magnitude across specifications, supporting the assumption that missingness was at random conditional on observed variables and that our complete-case approach did not materially bias the findings.

A two-tailed significance threshold of *p* < 0.05 was used for all statistical tests and models throughout the analysis. All statistical analyses and visualizations were performed using Intercooled Stata v18 [60].

## 5. Conclusions

In line with previous studies, we demonstrated that elevated liver enzyme levels were closely linked to poorer outcomes in patients with NAFLD, suggesting that GOT and GPT levels with demographic and clinical factors serve as strong prognostic biomarkers. The fact that the differences in survival probabilities became more pronounced with time indicated that monitoring transaminase levels over the course of a patients’ illness could be crucial for identifying those at greater risk and for potentially adjusting treatment strategies to improve patient outcomes. These results underline the necessity for enhanced surveillance of patients with NAFLD, focusing particularly on demographics such as age and sex, the management of comorbid conditions, and the regular monitoring of liver-related biomarkers. Ultimately, these insights could guide clinical protocols aiming to improve the survival outcomes of individuals with NAFLD. A complex therapeutical approach that includes effective weight management, lifestyle changes, and monitoring metabolic and cardiovascular health is essential in mitigating these risks and improving patient outcomes.

## Figures and Tables

**Table 1:** Baseline characteristics of the study sample at start of follow-up (2012).

Variable Name	Categories	*n* (%)
Age groups	<45	1905 (38.99)
	45–64	2227 (45.58)
	65+	754 (15.43)
	Total	4886
Sex	Male	2218 (45.42)
	Female	2665 (54.58)
	Total	4883
GOT	Normal	464 (87.88)
	Elevated	64 (12.12)
	Total	528
GPT	Normal	489 (86.55)
	Elevated	76 (13.45)
	Total	565
C-reactive protein	Normal (≤15)	358 (81.74)
	High (>15)	80 (18.26)
	Total	438
Essential hypertension	absent	4274 (87.47)
	present	612 (12.53)
	Total	4886
Angina pectoris	absent	4550 (93.12)
	present	336 (6.88)
	Total	4886
Chronic ischemic heart disease	absent	4715 (96.50)
	present	171 (3.50)
	Total	4886
Heart failure	absent	4824 (98.73)
	present	62 (1.27)
	Total	4886
Atherosclerosis	absent	4790 (98.04)
	present	96 (1.96)
	Total	4886
Type 2 diabetes mellitus	absent	4785 (97.93)
	present	101 (2.07)
	Total	4886
Obesity	absent	4768 (97.58)
	present	118 (2.42)
	Total	4886
Disorders of lipid metabolism	absent	4592 (93.98)
	present	294 (6.02)
	Total	4886

Abbreviations: GOT, glutamic oxaloacetic transaminase (aspartate aminotransferase, AST); GPT, glutamic pyruvic transaminase (alanine aminotransferase, ALT); CRP, C-reactive protein. Cut-off values: GOT > 40 IU/L, GPT > 40 IU/L, CRP > 15 mg/L were considered elevated.

**Table 2:** Fleming–Harrington test results for survival distributions by covariates.

Variable	Group	Observed Events	Expected Events	Sum of Ranks	*p*-Value
**Age groups**	<45	62	91.91	−0.157	**0.012**
	45–64	190	164.73	0.108	
	65+	107	102.35	0.049	
**Sex**	Male	185	147.42	0.148	**0.014**
	Female	174	211.58	−0.148	
**GOT**	normal	74	92.59	−0.146	**<0.001**
	high	27	8.41	0.146	
**GPT**	normal	77	97.17	−0.175	**<0.001**
	high	34	13.83	0.175	
**C-reactive protein**	normal	46	53.22	−0.04	0.251
	high	22	14.78	0.04	
**Essential hypertension**	absent	278	340.08	−0.073	**0.007**
	present	81	18.92	0.073	
**Angina pectoris**	absent	309	347.43	−0.004	0.858
	present	50	11.57	0.004	
**Chronic ischemic heart disease**	absent	324	352.01	−0.046	**0.005**
	present	35	6.99	0.046	
**Heart failure**	absent	343	355	−0.048	**<0.001**
	present	16	4	0.048	
**Atherosclerosis**	absent	336	354.66	0.015	0.278
	present	23	4.34	−0.015	
**Type 2 diabetes mellitus**	absent	334	354.24	−0.039	**0.005**
	present	25	4.76	0.039	
**Obesity**	absent	328	355.13	−0.075	**<0.001**
	present	31	3.87	0.075	
**Disorders of lipid metabolism**	absent	278	347.9	−0.124	**<0.001**
	present	81	11.1	0.124	

**Footer**: The Fleming–Harrington test was conducted using parameters ρ = 0 and γ = 1, which assign slightly more weight to later events. Abbreviations: GOT, glutamic oxaloacetic transaminase (aspartate aminotransferase, AST); GPT, glutamic pyruvic transaminase (alanine aminotransferase, ALT); CRP, C-reactive protein. Significant *p*-values (*p* < 0.05) are highlighted in bold.

**Table 3:** Unadjusted restricted mean survival time (RMST) estimates for all covariates in the model.

Variable	Group	RMST Estimate	95% CI (Lower–Upper)	95% CI (Lower–Upper)	*p*-Value	Ratio (Group 1/0)	95% CI (Lower–Upper)	*p*-Value
Age groups	<45	6.966	6.959–6.972					
	45–64	6.944	6.938–6.950	−0.031–−0.013	**<0.001**	0.997	0.996–0.998	**<0.001**
	65+	6.952	6.946–6.959	−0.023–−0.004	**0.004**	0.998	0.997–0.999	**0.004**
Sex	Male	6.939	6.933–6.945	0.014–0.030	**<0.001**	1.003	1.002–1.004	**<0.001**
	Female	6.961	6.957–6.966					
GOT	normal	6.933	6.921–6.945	−0.352–−0.142	**<0.001**	0.964	0.949–0.980	**<0.001**
	high	6.686	6.582–6.791					
GPT	normal	6.936	6.923–6.948	−0.211–−0.087	**<0.001**	0.978	0.970–0.987	**<0.001**
	high	6.787	6.726–6.847					
C-reactive protein	normal	6.947	6.934–6.960	−0.103–−0.006	**0.027**	0.992	0.985–0.999	**0.027**
	high	6.893	6.846–6.939					
Essential hypertension	absent	6.964	6.960–6.968	−0.241–−0.199	**<0.001**	0.968	0.965–0.971	**<0.001**
	present	6.744	6.723–6.765					
Angina pectoris	absent	6.96	6.956–6.964	−0.259–−0.216	**<0.001**	0.966	0.963–0.969	**<0.001**
	present	6.723	6.701–6.744					
Chronic ischemic heart disease	absent	6.957	6.953–6.961	−0.282–−0.208	**<0.001**	0.965	0.959–0.970	**<0.001**
	present	6.712	6.675–6.749					
Heart failure (tau = 5)	absent	4.974	4.972–4.976	−0.107–−0.050	**<0.001**	0.984	0.978–0.990	**<0.001**
	present	4.895	4.866–4.924					
Atherosclerosis (tau = 3)	absent	2.99	2.990–2.991	−0.111–−0.110	**<0.001**	0.963	0.963–0.963	**<0.001**
	present	2.88	2.880–2.880					
Type 2 diabetes mellitus	absent	6.956	6.952–6.959	−0.322–−0.204	**<0.001**	0.962	0.954–0.971	**<0.001**
	present	6.693	6.634–6.752					
Obesity	absent	6.957	6.953–6.960	−0.489–−0.323	**<0.001**	0.942	0.930–0.954	**<0.001**
	present	6.551	6.467–6.634					
Disorders of lipid metabolism	absent	6.965	6.962–6.969	−0.461–−0.363	**<0.001**	0.941	0.934–0.948	**<0.001**
	present	6.553	6.504–6.602					

Abbreviations: RMST, restricted mean survival time; CI, confidence interval; GOT, glutamic oxaloacetic transaminase; GPT, glutamic pyruvic transaminase. Tau represents the maximum follow-up time for calculating RMST, which was set at 7 years, except for heart failure (tau = 5) and atherosclerosis (tau = 3). The “Ratio (Group 1/0)” represents the relative RMST between the exposed group (coded as 1) and the reference group (coded as 0). A ratio below 1 indicates shorter average survival time until NAFLD diagnosis in the exposed group compared to the reference group. Significant *p*-values (*p* < 0.05) are highlighted in bold.

**Table 4:** Flexible parametric survival model estimates.

Variable Name	Categories	HR [95% CI]	*p*-Value
Age groups	<45 (ref)		
	45–64	0.98 [0.68–1.40]	0.914
	65+	1.05 [0.64–1.74]	0.838
Sex	Male (ref)		
	Female	1.05 [0.64–1.74]	0.838
GOT	Normal (ref)		
	Elevated	**2.71 [1.31–5.58]**	**0.007**
GPT	Normal (ref)		
	Elevated	**2.21 [1.09–4.43]**	**0.027**
C-reactive protein	Normal (≤15, ref)		
	High (>15)	1.20 [0.70–2.08]	0.511
Essential hypertension	Absent (ref)		
	Present	1.70 [0.79–3.63]	0.173
Angina pectoris	Absent (ref)		
	Present	1.09 [0.32–3.72]	0.892
Chronic ischemic heart disease	Absent (ref)		
	Present	1.04 [0.32–3.41]	0.943
Heart failure	Absent (ref)		
	Present	0.77 [0.23–2.63]	0.68
Atherosclerosis	Absent (ref)		
	Present	1.78 [0.41–7.69]	0.441
Type 2 diabetes mellitus	Absent (ref)		
	Present	1.55 [0.52–4.66]	0.434
Obesity	Absent (ref)		
	Present	2.46 [0.83–7.24]	0.106
Disorders of lipid metabolism	Absent (ref)		
	Present	**3.29 [1.51–7.25]**	**0.003**
Nonlinear Effect 1 (_rcs1)	-	**1.43 [1.32–1.57]**	**<0.001**
Nonlinear Effect 2 (_rcs2)	-	**1.06 [1.00–1.12]**	**0.039**

Abbreviations: GOT—glutamic oxaloacetic transaminase, GPT—glutamic pyruvic transaminase, CI—confidence interval, _rcs1/_rcs2—restricted cubic spline nonlinear terms. Significant values (*p* < 0.05) are highlighted.

## Data Availability

The data presented in this study are available upon request from the corresponding author. The data are not publicly available due to institutional restrictions.
